# Co-expression Pattern Analysis of miR-17-92 Target Genes in Chronic Myelogenous Leukemia

**DOI:** 10.3389/fgene.2016.00167

**Published:** 2016-09-21

**Authors:** Fengfeng Wang, Fei Meng, Lili Wang

**Affiliations:** Department of Health Technology and Informatics, Hong Kong Polytechnic UniversityHong Kong, China

**Keywords:** microRNA, co-expression, disease-specific cutoff point, metabolism, chronic myelogenous leukemia

## Abstract

MicroRNAs (miRNAs) are post-transcriptional regulators that regulate gene expression by binding to the 3′ untranslated region of target mRNAs. Mature miRNAs transcribed from the miR-17-92 cluster have an oncogenic activity, which are overexpressed in chronic-phase chronic myelogenous leukemia (CML) patients compared with normal individuals. Besides, the tyrosine kinase activity of BCR-ABL oncoprotein from the Philadelphia chromosome in CML can affect this miRNA cluster. Genes with similar mRNA expression profiles are likely to be regulated by the same regulators. We hypothesize that target genes regulated by the same miRNA are co-expressed. In this study, we aim to explore the difference in the co-expression patterns of those genes potentially regulated by miR-17-92 cluster between the normal and the CML groups. We applied a statistical method for gene pair classification by identifying a disease-specific cutoff point that classified the co-expressed gene pairs into strong and weak co-expression classes. The method effectively identified the differences in the co-expression patterns from the overall structure. Functional annotation for co-expressed gene pairs showed that genes involved in the metabolism processes were more likely to be co-expressed in the normal group compared to the CML group. Our method can identify the co-expression pattern difference from the overall structure between two different distributions using the distribution-based statistical method. Functional annotation further provides the biological support. The co-expression pattern in the normal group is regarded as the inter-gene linkages, which represents the healthy pathological balance. Dysregulation of metabolism may be related to CML pathology. Our findings will provide useful information for investigating the novel CML mechanism and treatment.

## Introduction

Chronic myelogenous leukemia (CML) is a clonal myeloproliferative disorder of the hematopoietic stem cells (Salesse and Verfaillie, 2002). The hallmark of CML is the Philadelphia (Ph) chromosome, which results from a balanced reciprocal translocation event t(9;22)(q34;q11) between chromosome 9 and 22 (Nowell and Hungerford, 1960; Rowley, 1973). Fusion BCR-ABL oncoprotein is produced by *BCR-ABL* oncogene, which combines the Abelson oncogene (*ABL*) at 9q34 and the breakpoint cluster region (*BCR*) at 22q11.2 through this translocation (Melo and Barnes, 2007). Such fusion can increase the tyrosine kinase activity of ABL and the autophosphorylation of BCR-ABL oncoprotein, creating more binding sites for the interacting proteins (Melo and Barnes, 2007).

Recently, more microarray studies have been performed in CML, such as the gene differential and co-expression analysis. The differential expression analysis can only identify the upregulation or downregulation of genes, which cannot reflect the functional linkages among genes during signal transduction. The co-expression analysis is very powerful for grouping genes and further analyzing the underlying mechanisms of diseases. In addition, gene co-expression patterns vary among different states and cell types (Torkamani et al., 2010). Hence, the altered co-expression pattern can be served as the signature of a disease. It was reported that genes with similar mRNA expression profiles tend to be regulated by the same mechanism(s), e.g., sharing common regulator (Altman and Raychaudhuri, 2001; Schulze and Downward, 2001).

MicroRNAs (miRNAs) are emerging as a new class of gene regulatory factors regulating human gene expression during the post-transcriptional process. MiRNAs are short and noncoding RNA molecules, with about 22 nucleotides long, which can bind to the complementary sequences in the 3′ untranslated region (3′UTR) of mRNAs (Kumar et al., 2007). MiRNAs are also found to be involved in multiple steps of myeloid differentiation, for example, the differentiation of common progenitor from the early stage to the terminal stage (Bhagavathi and Czader, 2010). The miR-17-92 cluster located in chromosome 13 transcribes to 7 mature miRNAs (miR-17-5p, miR-17-3p, miR-18a, miR-19a, miR-20a, miR-19b, and miR-92-1) (Coller et al., 2007; Aguda et al., 2008). It is worth mentioning that these 7 mature miRNAs have similar expression patterns in hematopoietic cell lines (Yu et al., 2006; Coller et al., 2007). Expressions of these miRNAs were found to promote cell proliferation, inhibit apoptosis, and induce tumor angiogenesis in cancer cells (Mendell, 2008). Moreover, this cluster is overexpressed in chronic-phase CML patients compared to normal individuals, and its overexpression can promote cell cycle progression and proliferation, and inhibit apoptosis (Venturini et al., 2007; Mendell, 2008). Thus, the BCR-ABL tyrosine kinase activity may affect the functions of this miRNA cluster.

In this study, we hypothesize that target genes regulated by the same miRNA should be co-expressed. We explored the difference in the co-expression patterns of those target genes potentially regulated by miR-17-92 cluster between the normal and the CML groups. We applied a statistical method to identify a disease-specific cutoff point for co-expression levels that grouped the co-expressed gene pairs into strong and weak co-expression classes so that one of the classes was best coherent with the CML state (Wang et al., 2014a, 2015a,b; Chan et al., 2015). Previous co-expression analysis calculates a *p*-value of correlation coefficient for each gene pair to identify significantly co-expressed gene pairs. Our method formed two distributions based on all the correlation coefficients of gene pairs in two different groups. By analyzing the biological meaning of strongly co-expressed gene pairs, we can further explore the underlying mechanisms of CML, and provide useful information for cancer treatment.

## Methods

### Microarray expression data

In this study, we chose the microarray dataset GSE5550, which is publicly available in the *Gene Expression Omnibus* (*GEO*) repository database (Diaz-Blanco et al., 2007). The data are normalized by variance stabilizing transformations (VSN) method across the samples. This dataset was obtained from gene expression measurements of 8537 unique mRNAs. CD34 + hematopoietic stem and progenitor cells were collected from bone marrows of untreated chronic-phase CML patients and health controls (Diaz-Blanco et al., 2007). The subjects recruited were Caucasians from Germany. Two groups of sample are included in this dataset: (i) the CML group: nine patients; and (ii) the control group: eight normal individuals. For microarray data, a gene may be interrogated by more than one probe. The average of all the probes for the same mRNA was taken to deal with this situation (Breslin et al., 2005; Kapp et al., 2006).

### Identification of candidate target genes potentially regulated by miR-17-92 cluster

The systematic search for genes targeted by miR-17-92 cluster (miR-17-5p, miR-17-3p, miR-18a, miR-19a, miR-20a, miR-19b and miR-92-1) was performed on five miRNA prediction databases (*DIANA-microT, MicroCosm-Targets, miRWalk, TargetScan* and *miRDB*). Some prediction databases (e.g., *DIANA-microT* and *TargetScan*) predict the miRNA targets based on three basic criteria: (i) complementarity when miRNAs bind to mRNAs in seed regions;(ii) free energy to fold the miRNA-mRNA duplex; and (iii) conservation among different species (Li et al., 2010; Chan et al., 2012; Wang et al., 2014b). In the first step, we obtained the target genes regulated by each mature miRNA from the miR-17-92 cluster using these five prediction databases. In order to increase the prediction accuracy, we selected the mRNAs predicted by at least four out of five databases. In the next step, we combined the target genes from all the seven mature miRNAs as the candidate target genes potentially regulated by miR-17-92 cluster for the following co-expression analysis.

### Co-expression analysis for candidate target genes

#### Co-expression measure for gene pairs

Pearson correlation coefficient (r) was chosen as the similarity measure to calculate the correlation coefficients of gene pairs in this study. Pearson correlation coefficient is represented by the direction cosine between two vectors normalized by the subtraction of their own means. Generally, similarity measure is regarded as a kernel function between two feature vectors. In this study, each feature vector contained the expression profiles of a gene across all the samples in the normal or the CML group respectively. The absolute values of correlation coefficients (|r| values) were considered, due to that the co-expression measure output a scalar in the range from 0 to 1 where a high output indicated a strong biological relationship in either positive or negative direction, and a low output represented a weak biological relationship. The co-expression level was denoted by *C*_*d*_*(i, j)* if the expression profiles of two genes were extracted from the disease (CML) group, and *C*_*n*_*(i, j)* for the normal group, as shown in Formulas 1 and 2.

(1) $$\begin{array}{r} C_{d}\left(i,j\right)=|cor\left(x_{di},x_{dj}\right)| \end{array}$$

(2) $$\begin{array}{r} C_{n}\left(i,j\right)=|cor\left(x_{ni},x_{nj}\right)| \end{array}$$

where *C*_*d*_*(i, j)* and *C*_*n*_*(i, j)* refer to the absolute values of correlation coefficients between the expression profiles of gene *i* and gene *j* in the CML group and the normal group, respectively (Horvath and Dong, 2008); *x*_*di*_ and *x*_*dj*_ represent the expression profiles of the *i*^*th*^ and *j*^*th*^ genes in the CML group; *x*_*ni*_ and *x*_*nj*_ refer to the expression profiles of the *i*^*th*^ and *j*^*th*^ genes in the normal group; *cor(x*_*di*_*, x*_*dj*_*)* stands for the Pearson correlation coefficient between the *i*^*th*^ and *j*^*th*^ genes in the CML group; *cor(x*_*ni*_*, x*_*nj*_*)* represents the Pearson correlation coefficient between the *i*^*th*^ and *j*^*th*^ genes in the normal group.

#### Identification of disease-specific cutoff point

Two sets of correlation coefficients in the normal and CML groups were obtained. These two sets of data formed two different cumulative distributions. In the next step, we performed two-sample Kolmogorov-Smirnov (KS) test to exam if these two sets of correlation coefficients significantly differed in terms of the overall distributions between two different conditions. The significance for KS test was represented by comparing the the maximum deviation between two cumulative distributions of *C*_*d*_ and *C*_*n*_ (Formulas 3-5) to a critical *D* value (*D*_*critical*_) based on our previous developed method (Chan et al., 2015). At the maximum deviation a threshold was identified to classify the co-expressed gene pairs into strong and weak co-expression classes, called the disease-specific cutoff point (*C*), so that the class was significantly associated with the CML state. The cutoff point represented a co-expression level, at which *F*_*d*_ and *F*_*n*_ were extremely deviated.

(3) $$\begin{array}{r} D=\underset{C}{\max}|F_{d}\left(C\right)-F_{n}\left(C\right)| \end{array}$$

(4) $$\begin{array}{r} F_{d}\left(C\right)=Prob\left(C_{d}\geq C\right) \end{array}$$

(5) $$\begin{array}{r} F_{n}\left(C\right)=Prob\left(C_{n}\geq C\right) \end{array}$$

where *F*_*d*_ and *F*_*n*_ represent the cumulative distribution functions (CDFs) of *C*_*d*_ and *C*_*n*_, respectively; *D* is defined as the maximum deviation; *C* represents the disease-specific cutoff point.

#### Classification of co-expressed gene pairs

After the disease-specific cutoff point was identified, the gene pairs were classified into four co-expression classes according to the distributions: (i) strongly co-expressed gene pairs in the normal group: with |r| values bigger than or equal to *C* in the normal group; (ii) strongly co-expressed gene pairs in the CML group: with |r| values bigger than or equal to *C* in the CML group; (iii) weakly co-expressed gene pairs in the normal group: with |r| values smaller than *C* in the normal group; and (iv) weakly co-expressed gene pairs in the CML group: with |r| values smaller than *C* in the CML group.

For better illustration of the groups' characteristics, we further identified the specifically co-expressed gene pairs to form the co-expression galaxy. The normal-specific strongly co-expressed pairs were the gene pairs strongly co-expressed only in the normal group, which were regarded as the inter-gene linkages maintaining physiological balance in healthy individuals. Apparently, these pairs were the CML-specific weakly co-expressed pairs, which were weakly co-expressed only in the CML group. The CML-specific strongly co-expressed pairs were the gene pairs strongly co-expressed only in the CML group, which represented the characteristics of the disease and may be the pathogenic alternatives. Similarly, these pairs were served as the normal-specific weakly co-expressed pairs.

### Functional annotation for candidate target genes

Gene ontology (GO) provides a systematic language and concept collection to describe genes and their product attributes across all species (Gene Ontology Consortium, 2008). In this study, we applied biological process of gene ontology to annotate the candidate target genes potentially regulated by miR-17-92 cluster, to further explore the biological meaning for the identified co-expressed gene pairs. *Database for Annotation, Visualization and Integrated Discovery* (*DAVID*) was applied to perform the functional annotation (Huang da et al., 2009). Functional annotation chart was chosen to select the significant batch annotation and GO terms that were most pertinent to the input data when the candidate target gene list was uploaded to *DAVID*. The significance of GO term enrichment is calculated based on a modified Fisher's Exact Test with Expression Analysis Systematic Explorer (EASE) score. Using *DAVID*, we annotated the candidate target genes involved in the significantly associated GO terms for a set of biological processes. The selection criteria for the significant GO terms were: (i) EASE score < 0.05; and (ii) false discovery rate (FDR) < 0.05, for multiple-hypothesis correction. Candidate target genes identified in each significant GO term were called the annotated target genes.

### Mapping co-expressed gene pairs to annotated gene pairs

The annotated target genes in each GO term were paired with all the possible combinations to form the annotated gene pairs. In the next step, the annotated gene pairs were mapped to the identified co-expressed gene pairs: the mapped normal-specific strongly, the mapped normal-specific weakly, the mapped CML-specific strongly and the mapped CML-specific weakly co-expressed pairs. Fisher exact test was used to identify if there were more mapped normal-specific strongly co-expressed pairs than mapped CML-specific strongly co-expressed pairs in each GO term for biological process. Therefore, one-sided *p*-value was chosen to indicate the significance. The multiple-hypothesis correction for the whole set of significant GO terms for biological processes was performed by following a more stringent method, Bonferroni correction. That is, the *p*-value of each GO term was multiplied by the total number of considered GO terms to correct the *p*-value. A GO term was significantly mapped if its corrected *p*-value was still smaller than the error rate (0.05).

## Results

### Identification of overall structural difference in co-expression

**Figure 1:**
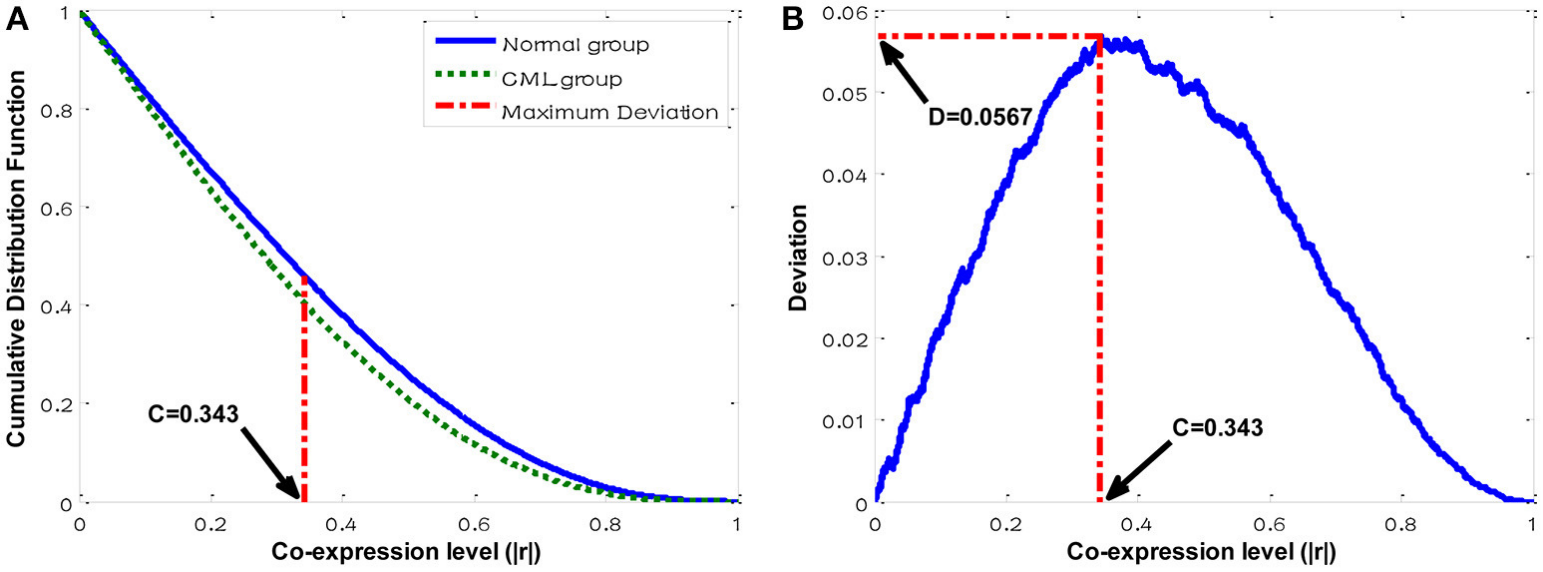
**Distribution plots for the co-expression analysis. (A)** Cumulative distributions of co-expression levels in the normal and the CML groups. **(B)** Deviation distribution against different co-expression cutoff points.

The candidate target genes potentially regulated by miR-17-92 cluster were collected from the five prediction databases. In order to perform the co-expression analysis, the target genes should be found in the microarray dataset. In all, we identified 288 candidate target genes in the microarray dataset GSE5550 (Table S1). We further extracted the available expression profiles of these 288 genes and calculated the correlation coefficients in the normal group and the CML group, respectively, forming the correlation coefficients of 41,328 gene pairs in each group. The cumulative distributions for these two sets of data were plotted. Two-sample KS test was used to identify the difference from the overall structure. We found that these two distributions in the normal and CML groups were significantly different with *p* < 0.05 for the maximum deviation *D* = 0.0567 > *D*_*critical*_ = 0.009 (Figure 1). The disease-specific cutoff point, *C* = 0.343, was identified at the maximum deviation (Figure 1). The cutoff point grouped gene pairs into four co-expression classes based on the co-expression levels (Table 1). We can infer that these two co-expression patterns were so distinct that the normal group had more strongly co-expressed (level above ~0.343) and less weakly co-expressed (level below ~0.343) gene pairs compared to the CML group. These candidate target genes tended to be co-expressed in the normal group when compared to the CML group.

**Table 1:** **Gene pair counts identified by the disease-specific cutoff point**.

**Group**	**No. of strongly co-expressed gene pairs**	**No. of weakly co-expressed gene pairs**
Normal	18,999	22,329
CML	16,654	24,674

### Co-expression galaxy and structures for the candidate target genes potentially regulated by miR-17-92 cluster

**Figure 2:**
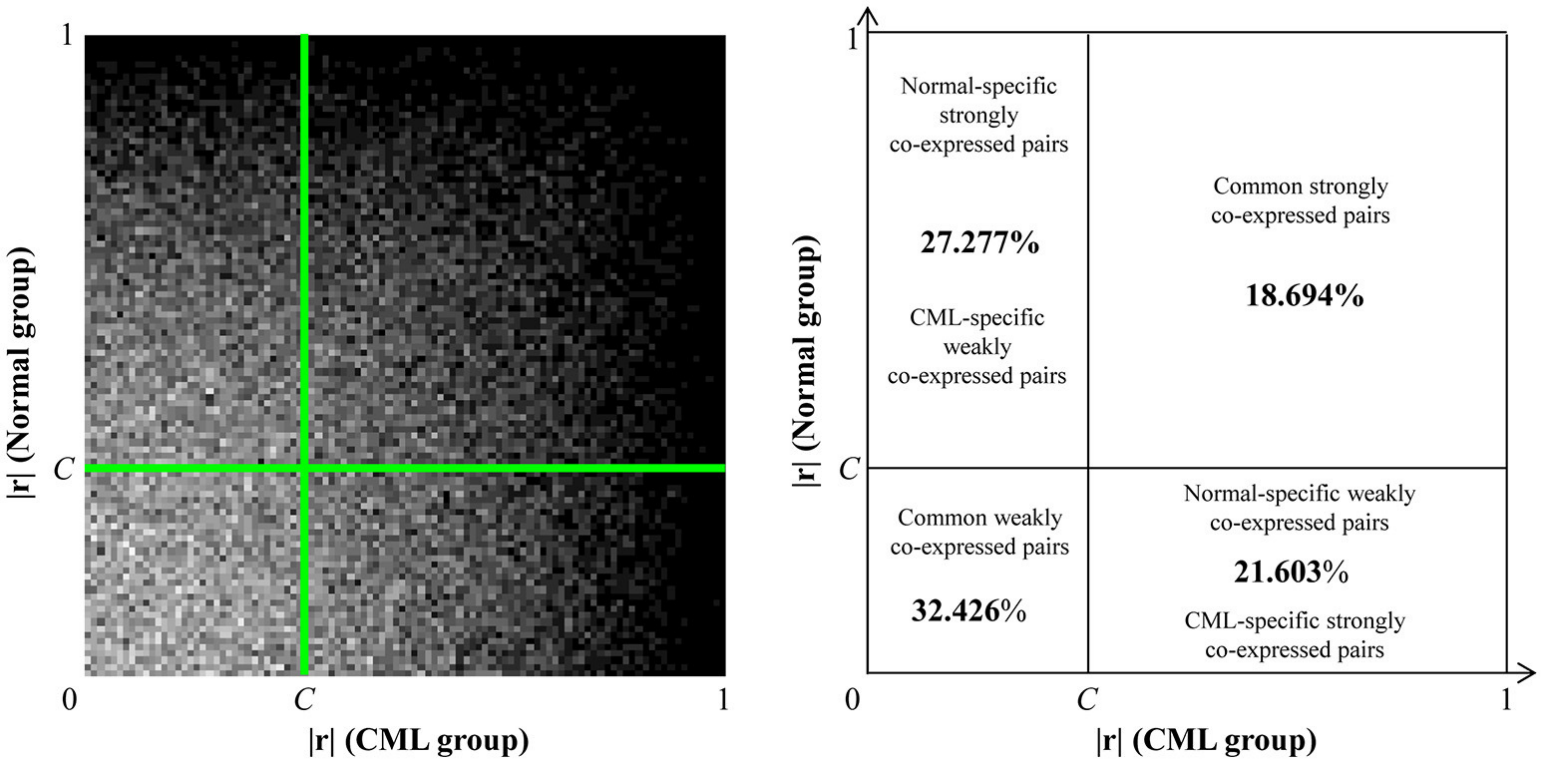
**Co-expression galaxy (left) and four regions partitioned by the disease-specific cutoff point, ***C*** = 0.343 (right)**. Each correlation coefficient (|r|) is represented by one white dot in the galaxy. More dots mean that there are more correlation coefficients located in that region.

The co-expression galaxy was plotted and partitioned into four regions: (i) normal-specific strongly co-expressed pairs (CML-specific weakly co-expressed pairs): the percentage was 27.277%; (ii) common strongly co-expressed pairs: the percentage was 18.694%; (iii) CML-specific strongly co-expressed pairs (normal-specific weakly co-expressed pairs): the percentage was 21.603%; and (iv) common weakly co-expressed pairs: the percentage was 32.426% (Figure 2). From the results, we observed that there were more normal-specific strongly co-expressed pairs than CML-specific strongly co-expressed pairs.

### DAVID annotation for enriched gene ontology terms

Based on the selection criteria (EASE score < 0.05 and FDR < 0.05), 11 significant GO terms for biological processes were found (Table 2). We obtained the annotated target genes from each biological process and formed the annotated gene pairs. After that, the co-expressed gene pairs were mapped to the annotated gene pairs. The results revealed that all these 11 biological processes had more mapped normal-specific strongly co-expressed pairs than mapped CML-specific strongly co-expressed pairs (Table 3). Fisher exact test was applied to indicate the significance. Eight of 11 biological processes had significant fisher exact test *p*-values (*p* < 0.05) and the corrected *p*-values for multiple-hypothesis correction was also smaller than 0.05: *Positive regulation of nitrogen compound metabolic process, Positive regulation of nucleobase, nucleoside, nucleotide and nucleic acid metabolic process, Positive regulation of biosynthetic process, Positive regulation of cellular biosynthetic process, Positive regulation of macromolecule biosynthetic process, Positive regulation of transcription, DNA-dependent, Positive regulation of RNA metabolic process* and *Positive regulation of cellular metabolic process*. From the results, we observed that most of the processes were related to metabolism, including nitrogen compound metabolic process, cellular biosynthetic process and RNA metabolic process. Moreover, all these eight significant biological processes perform the positive regulation function. Our results demonstrated that genes involved in these processes tended to be more co-expressed in the normal group when compared to the CML group. In other words, the co-expression pattern was dysregulated in CML.

**Table 2:** **Enriched biological process GO terms for functional annotation of candidate target genes**.

**No**.	**Significant GO terms**	**Genes found in our data**	**EASE score**	**FDR**
1	Positive regulation of nitrogen compound metabolic process	37	4.50 × 10^−08^	7.30 × 10^−05^
2	Positive regulation of nucleobase, nucleoside, nucleotide and nucleic acid metabolic process	36	6.50 × 10^−08^	1.10 × 10^−04^
3	Positive regulation of biosynthetic process	38	1.00 × 10^−07^	1.60 × 10^−04^
4	Positive regulation of cellular biosynthetic process	37	2.10 × 10^−07^	3.40 × 10^−04^
5	Positive regulation of transcription	32	6.20 × 10^−07^	1.00 × 10^−03^
6	Positive regulation of macromolecule biosynthetic process	35	6.20 × 10^−07^	1.00 × 10^−03^
7	Positive regulation of gene expression	32	1.20 × 10^−06^	1.90 × 10^−03^
8	Positive regulation of transcription, DNA-dependent	28	2.00 × 10^−06^	3.30 × 10^−03^
9	Positive regulation of RNA metabolic process	28	2.30 × 10^−06^	3.80 × 10^−03^
10	Positive regulation of cellular metabolic process	40	4.50 × 10^−06^	7.30 × 10^−03^
11	Positive regulation of macromolecule metabolic process	38	1.50 × 10^−05^	2.40 × 10^−02^

GO, gene ontology; EASE score, Expression Analysis Systematic Explorer score (a modified Fisher's Exact Test); FDR, false discovery rate.

**Table 3:** **Mapping co-expressed gene pairs to annotated gene pairs from each biological process GO term**.

**GO terms**	**Fisher exact test**					**Corrected *p*-value**
	***a***	***b***	***c***	***d***	***p*-value**	
**Positive regulation of nitrogen compound metabolic process**	186	148	148	186	**0.002**	**0.022**
**Positive regulation of nucleobase, nucleoside, nucleotide and nucleic acid metabolic process**	177	141	141	177	**0.003**	**0.033**
**Positive regulation of biosynthetic process**	196	156	156	196	**0.002**	**0.022**
**Positive regulation of cellular biosynthetic process**	192	146	146	192	<0.001	<0.001
Positive regulation of transcription	137	111	111	137	0.012	0.132
**Positive regulation of macromolecule biosynthetic process**	169	129	129	169	**0.001**	**0.011**
Positive regulation of gene expression	137	111	111	137	0.012	0.132
**Positive regulation of transcription, DNA-dependent**	110	79	79	110	**0.001**	**0.011**
**Positive regulation of RNA metabolic process**	110	79	79	110	**0.001**	**0.011**
**Positive regulation of cellular metabolic process**	216	170	170	216	**0.001**	**0.011**
Positive regulation of macromolecule metabolic process	188	153	153	188	0.005	0.055

The GO terms highlighted with bold text are significantly mapped; a, mapped normal-specific strongly co-expressed pairs; b, mapped normal-specific weakly co-expressed pairs; c, mapped CML-specific strongly co-expressed pairs; d, mapped CML-specific weakly co-expressed pairs.

## Discussion

In this study, we have successfully identified the overall differences in the co-expression patterns of those candidate target genes potentially regulated by miR-17-92 cluster between the normal and the CML groups. Two-sample KS test was performed to indicate the difference (Figure 1). In the first step, the maximum deviation between two cumulative distributions revealed the difference structurally. After that, a disease-specific cutoff point was identified at the maximum deviation to group the co-expressed gene pairs so that the class was best coherent with the CML disease. We further identified the specifically co-expressed gene pairs in different groups to explore the alterations of biological processes.

The functional annotation from *DAVID* database showed that genes related to metabolism were more likely to be co-expressed in the normal group compared to the CML group (Table 3). Dysregulated mRNA metabolism is regarded as a feature for many human cancers, including CML (Perrotti and Neviani, 2007). BCR/ABL oncoprotein was found to affect the basal mRNA translation machinery by regulating the function of translation factors eukaryotic translation initiation factor 4E and its binding protein (Perrotti and Neviani, 2007). Other researchers reported that the metabolic patterns of untreated CML patients were different from healthy controls, which indicated the metabolic dysregulation in CML patients (Jiye et al., 2010). In addition, CML patients had lower levels of tricarboxylic acid cycle and lipid metabolism when compared to health individuals (Pelicano et al., 2006; Denkert et al., 2008; Jiye et al., 2010) In our study, all these significantly mapped biological processes were found to perform the positive regulation function (Table 3). However, the positive function was dysregulated in the CML group that there were less strongly co-expressed gene pairs found in the CML group compared to the normal group.

Researchers found that genes with similar mRNA expression profiles tend to be regulated by the same mechanism(s), e.g., the same regulator (Altman and Raychaudhuri, 2001; Schulze and Downward, 2001). MiRNA is a post-transcriptional regulator. In this study, we hypothesize that target genes regulated by the same miRNA should be co-expressed. The originality of our study is the application of structural co-expression analysis for the miR-17-92 cluster target genes to identify the different co-expression patterns between the normal and the CML states. The miRNA targets predicted by at least four out of five prediction databases were considered in our study, which made the prediction accuracy more reliable. Some of the predicted targets have been validated by other researchers. MiR-18a targets Dicer (DICER1) in two binding sites, and both sites could suppress expression using luciferase reporter assay *in vitro* (Tao et al., 2012). MiR-19a and 19b directly target N-Myc (MYCN), and could suppress the endogenous protein expression in a neuroblastoma cell line (Buechner et al., 2011). MiR-20a can down-regulate STAT3 protein expression and inhibit cell proliferation and invasion in pancreatic carcinoma (Yan et al., 2010).

Compared to the gene differential expression analysis, co-expression analysis is more useful to identify the functionally associated linkages among genes during signal transduction. In addition, gene co-expression analysis takes into account the level of correlations that may exist between gene expression patterns (Torkamani et al., 2010). Hence, the gene co-expression analysis is usually used to analyze the underlying mechanisms of diseases. Moreover, the different co-expression pattern can be served as a signature for the disease.

In this study, we presented a method to group the co-expressed gene pairs into strong and weak co-expression classes by identifying a disease-specific cutoff point to form the co-expression galaxy. This method was further applied to explore the differences in the co-expression patterns of those candidate target genes potentially regulated by miR-17-92 cluster. The co-expression pattern differences between the normal and the CML groups were identified from the overall structure. The different co-expression pattern can reflect the biological alterations in the CML state. We also found the dysregulated metabolism processes in CML. Our developed method and significant findings will provide useful information for cancer treatment.

## Author contributions

FW initiated the project and participated in its design. FW designed the co-expression analysis method and gene ontology annotation, and performed the analyses. FM and LW participated in the design and coordination of the study. FW was responsible for writing the manuscript. All the authors participated in discussion and editing of the manuscript.

### Conflict of interest statement

The authors declare that the research was conducted in the absence of any commercial or financial relationships that could be construed as a potential conflict of interest.
